# Trends of leprosy and multibacillary infection in the state of Georgia since the early 1900s

**DOI:** 10.1371/journal.pntd.0007713

**Published:** 2019-10-11

**Authors:** Carter D. McCormick, Jacqueline Lea, Barbara M. Stryjewska, Ashton Thompson, Jessica K. Fairley

**Affiliations:** 1 Hubert Department of Global Health, Emory University Rollins School of Public Health Atlanta, GA, United States of America; 2 National Hansen’s Disease Programs, Baton Rouge, LA, United States of America; 3 Georgia Department of Public Health, Atlanta, GA, United States of America; Hospital Infantil de Mexico Federico Gomez, UNITED STATES

## Abstract

Few investigations to date have analyzed the epidemiology of Hansen’s disease (leprosy) in the United States, and in particular, if birth location is related to multibacillary versus paucibacillary leprosy. We collected data on 123 patients diagnosed with leprosy in Georgia from the National Hansen’s Disease Program from 1923—January 2018. A logistic regression model was built to examine the relationship between country of origin (U.S.-born or immigrant) and the type of leprosy. While the model showed no significant relationship between country of origin and type of leprosy, being Asian or Pacific Islander was associated with a higher odds of multibacillary disease (aOR = 5.71; 95% CI: 1.25–26.29). Furthermore, since the early 1900s, we found an increasing trend of leprosy reports in Georgia among both domestic born and immigrant residents, despite the overall decrease in cases in the United States during the same time period. More research is therefore necessary to further evaluate risk for multibacillary leprosy in certain populations and to create targeted interventions and prevention strategies.

## Author summary

Leprosy is often believed to not be an issue in many countries. However, the disease is still very much a problem globally, and in the past several decades has been on the rise in the United States. Frequently the disease is misdiagnosed several times before appropriately treated. Although newly diagnosed cases in the United States only remain around 160 every year, there is limited investigation of leprosy in the United States. As the concern over transmission from armadillos continues to rise, coupled with the increase in connectedness of the global population, it is important to be able to identify populations at higher risk for the more infectious form of leprosy, multibacillary leprosy, for targeted interventions. The researchers sought to investigate which populations are at higher risk for this more infectious form, while also describing the history of cases in the state of Georgia. By adding to the body of literature on leprosy in the United States, it is the hope that more research will be produced that provides a better understanding of the disease trends within the country, while also enabling more targeted interventions.

## Introduction

Leprosy (also known as Hansen’s Disease) is an uncommon infectious disease that has been affecting people since ancient times. It is caused by the bacillus, *Mycobacterium leprae (M*. *leprae)*, which can affect the skin, peripheral nerves, mucosa of the upper respiratory tract, and the eyes[1]. It commonly presents as skin lesions, often with hypo- or hyperpigmentation combined with sensory loss[2]. Hansen’s disease can lead to permanent disability due to damage of the nerves and is historically associated with stigma and discrimination, which can lead to depression and social isolation in many cases[1].

Leprosy is classified by histopathology from a skin biopsy, or by clinical presentation when a biopsy is not available[2, 3]. In the United States, the Ridley-Jopling classification is used for diagnosis, assigning patients as having indeterminate, tuberculoid, borderline tuberculoid, mid-borderline, borderline lepromatous, or lepromatous leprosy[3]. The World Health Organization (WHO) classifies patients with less than five lesions as paucibacillary (PB) and those with five or more lesions as multibacillary (MB)[3]. Patients may also present with thickened peripheral nerves, associated with numbness, muscle weakness, or paralysis[2]. Transmission of *M*. *leprae* occurs through droplets from the nose and mouth during close and frequent contact with untreated patients, usually those with multibacillary infection[1]. Studies in recent years in the United States and Brazil have also established probable transmission from the nine-banded armadillo (*Dasypus novemcinctus*), which is the only other mammalian reservoir of *M*. *leprae* and the only known environmental reservoir within the Americas[4, 5]. Leprosy is treated with an intensive multidrug therapy (MDT) regimen for up to 12 months, depending on the classification[6].

Global leprosy burden affects low- and middle-income countries predominately, with about 200,000 cases reported annually[7]. India, Brazil, and Indonesia have the most cases reported annually, making up about half of those reported in 2016[8]. The number of cases reported from higher income countries tend to be much smaller, such as 5 cases for the Netherlands in 2016[8]. However, the United States sits at a slightly higher case number per year, with 168 cases reported in 2016 (5.2x10^-7^ per million people), which is consistent with the number of cases reported in prior years[8].

The United States has a long history of leprosy, even before its founding, tracing back to the Acadian people from Canada who migrated to French Louisiana in the mid 1700s[9]. In 1917, the United States Senate passed Senate Bill 4086, which created a National Leprosarium in Carville, Louisiana[10]. This leprosarium became the base of the National Hansen’s Disease Program (NHDP), whose center was later moved in 1998 to Baton Rouge, Louisiana, and continues to be the main clinical, epidemiologic, and diagnostic reference in the United States for leprosy today[10].

Currently, most of the cases of leprosy seen in the United States are among immigrants from countries with higher leprosy endemicity[11]. In fact, between 1978–1988, 90% of reported cases were among immigrants[11]. More recent data confirm this trend, with the incidence of leprosy among immigrants being 14 times higher than among domestic born citizens in 2011[12]. A chart review of leprosy patients at the Mayo Clinic in Rochester, MN showed 67% of the patients were immigrants[13]. Additionally, 86% of cases at the Emory Healthcare’s TravelWell Clinic, the Atlanta NHDP affiliated clinic, between 2002 to 2014 were immigrants[14]. Leprosy in U.S. born citizens is likely either acquired while traveling in a leprosy endemic country or through exposure to infected armadillos in the United States.[15–17].

There are very few studies that examine the differences in PB and MB leprosy among immigrants and U.S. born citizens. Because MB disease is associated with a higher risk of complications and secondary transmission, it is important to understand the patterns of disease manifestations and epidemiologic characterizations of this type of leprosy[7]. Whether one has MB disease is thought to be based on host predisposition, but no data exist about the patterns of disease presentation based on the potential type of transmission (zoonotic versus person-to-person)[18]. Additionally, studies have shown that overall MB is more common in the U.S. (between 60–68%), but there has been no direct comparison between the two populations (immigrant versus U.S. born) and the likelihood of being diagnosed with MB or PB[11, 19]. This is especially important in southern states where zoonotic transmission occurs and may be more likely associated with U.S. born cases, at least in recent years[4]. Therefore, this paper seeks to answer the question if location of birth is associated with MB or PB leprosy in Georgia, with the goal of better understanding the epidemiology of leprosy in a Southern U.S. state. While leprosy is not very contagious, with more than 95% of people being immune and thus not posing a public health threat in the US, given the potential disability of delayed diagnosis, decreased awareness of the disease on the part of many U.S. physicians, and the increased reports of armadillo reservoirs, it is important to better understand the transmission dynamics and epidemiology of the infection in a state with immigration and where armadillos are present. This, in turn, can lead to better-informed targeted interventions and prevention strategies. Given both the practice location in Georgia of select authors, their affiliations with state public health officials, and an inability to access data from other states, Georgia was chosen as the sole state of study for this manuscript.

## Methods

Data on age, ethnicity, location of birth, type of leprosy diagnosed, and sex were extracted from leprosy surveillance reports of cases in Georgia from 1923 to January 2018. This data came from the NHDP Center in Baton Rouge. Leprosy is a reportable disease in Georgia and is monitored primarily through passive surveillance. An example of the current surveillance form used can be seen in Appendix 1 (S1 Appendix). Inclusion into the study was any patient from any year on record who was a resident of Georgia or was reported by a clinic in Georgia.

Chi-squared tests and t-tests were done, where appropriate, to assess bivariate differences between the domestic and foreign-born populations. A multivariate logistic regression model was then built, with multibacillary (versus paucibacillary) leprosy as the outcome and birth location as the exposure of interest, controlling for several variables (age, sex, and ethnicity). Birth location was defined as the birth place of the patient, with domestic cases referring to patients who were born in one of the 50 U.S. states, and abroad locations referring to patients who were not born in the United States and displayed symptoms either before or after moving to the United States. Age and sex were included because MB is typically more common in older adults and men, respectively[11, 19, 20, 21, 22]. Ethnicity was included to control for individuals in subpopulations that may have a higher burden of multibacillary versus paucibacillary disease and vice versa, and included white, African American, Hispanic, Asian or Pacific Islander, and Indian or Middle Eastern[11]. A second model was built only using data from after 1995, when the WHO started providing MDT for all leprosy patients outside of the United States and Puerto Rico, to see if temporal changes impacted the relationship[22]. The ethnicity variable was modified due to the limited ethnic categories represented in the data after 1995. Interaction was assessed in both models.

This study was approved exempt from review by the Emory University Institutional Review Board. SAS version 9.4 (Cary, NC) was utilized for analysis.

## Results

**Fig 1:**
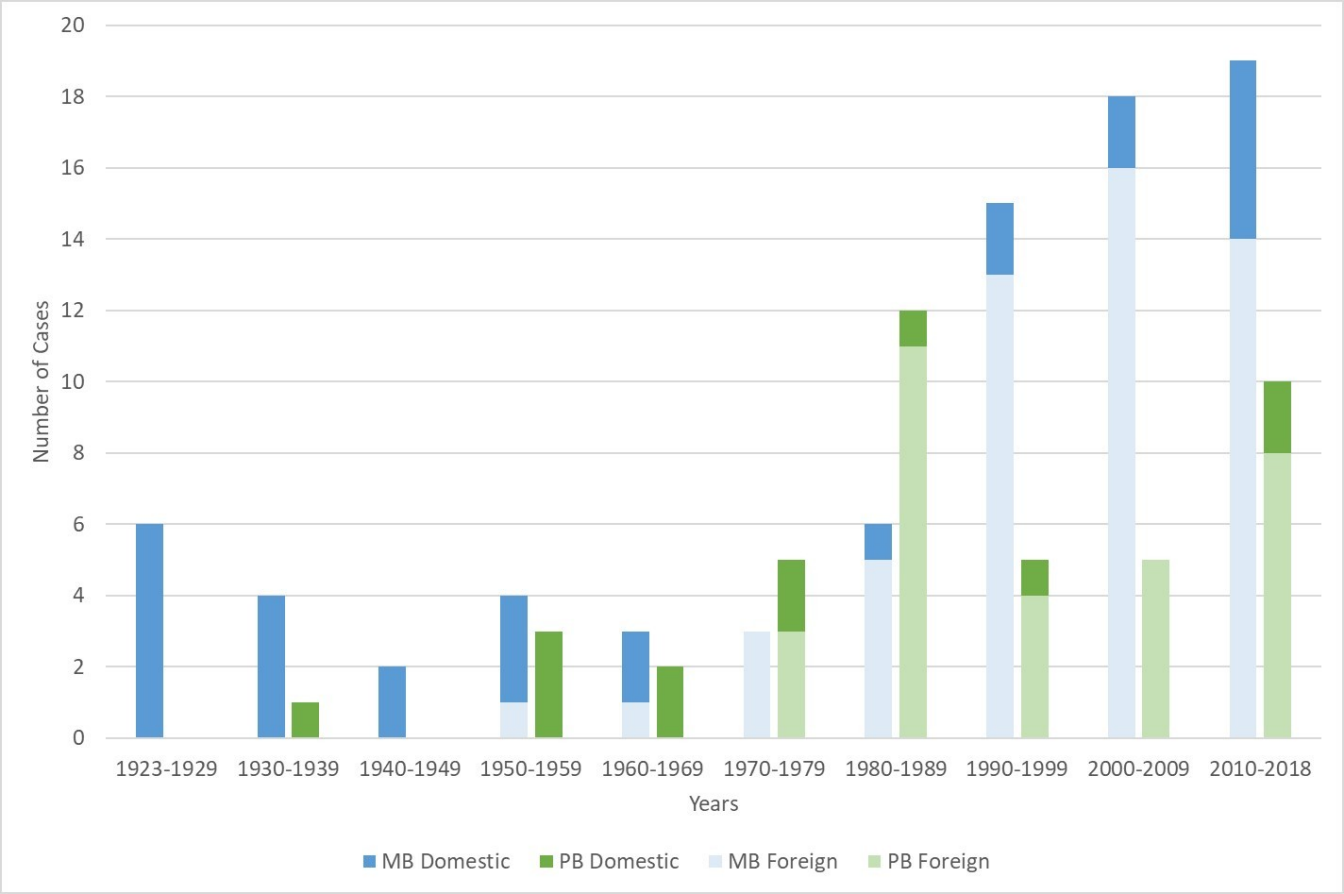
Number of leprosy cases in Georgia from 1923–2018 by birth location and leprosy type.

Of the 138 cases that met inclusion criteria at the National Hansen’s Disease Program, 123 cases (89.13%) were included in the final model for all years. The 15 cases not included had missing data for one or more of the variables and were thus excluded in this complete case analysis. Of the 123 cases, 39 (31.71%) were born domestically and 84 (68.29%) were born abroad (Table 1). For both groups, most had multibacillary leprosy (n = 27, 69.23% of those born domestically and n = 53, 63.10% of those born abroad) and the majority were male (n = 30, 76.92% of those born domestically and n = 54, 64.29% of those born abroad). The majority of cases born domestically were white (n = 27, 69.23%), while the largest ethnic group of cases born abroad were Asian or Pacific Islander (n = 37, 44.05%). A trend of increasing MB cases, and overall number of reported cases, started in the 1970s, most notably in foreign-born patients (Fig 1).

**Table 1:** Demographic characteristics of the 123 cases of leprosy in Georgia included in the study, divided by place of birth and evaluated by chi-square or t-test where appropriate.

	Born Domestic n = 39 (31.71%)	Born Abroad n = 84 (68.29%)	p-value
Leprosy Type, n (%)			
PB	12 (30.77)	31 (36.90)	0.5067^¥^
MB	27 (69.23)	53 (63.10)	
Sex, n (%)			
Female	9 (23.08)	30 (35.71)	0.161^¥^
Male	30 (76.92)	54 (64.29)	
Ethnicity, n (%)			
White	27 (69.23)	6 (7.14)	<0.0001^¥^
African American	10 (25.64)	10 (11.90)	
Hispanic	1 (2.56)	23 (27.38)	
Asian or Pacific Islander	1 (2.56)	37 (44.05)	
Indian or Middle Eastern	0 (0.00)	8 (9.52)	
Age, Mean (SD)	45.95 (14.24)	35.56 (13.74)	0.0002^†^

^†^p-value generated via t-test

^¥^p-value generated via chi-squared test

Location of birth was not significantly associated with the type of leprosy in the logistic regression model (aOR: 0.46; 95% CI: 0.12–1.80) when controlling for age, sex, and ethnicity (Table 2). However, being ethnically Asian or Pacific Islander versus white did have a significant relationship with MB leprosy (aOR: 5.74, 95% CI: 1.25–26.29) when controlling for age, sex, and location of birth. Interaction did not have an effect on the relationship for this model.

**Table 2:** Logistic regression model for the years 1923–2018.

	Univariate Analysis		Multivariate Analysis	
Parameter	OR (95% CI)	p-value	aOR (95%)	p-value
Birth location				
Domestic	1.0		1.0	
Abroad	1.32 (0.58–2.96)	0.507	0.46 (0.12–1.80)	0.261
Sex				
Female	1.0		1.0	
Male	0.68 (0.31–1.49)	0.338	0.70 (0.30–1.65)	0.417
Ethnicity				
White	1.0		1.0	
African American	1.68 (0.50–5.68)	0.683	1.86 (0.50–6.91)	0.995
Hispanic	0.82 (0.23–2.92)	0.237	1.31 (0.25–6.96)	0.477
Asian or Pacific Islander	3.86 (1.39–10.72)	0.004	5.74 (1.25–26.29)	0.004*
Indian or Middle Eastern	1.04 (0.17–6.22)	0.654	1.56 (0.18–13.18)	0.808
Age	0.98 (0.95–1.01)	0.110	0.98 (0.95–1.01)	0.156

* significant *p*-value at *p* < 0.05.

aOR = adjusted odds ratio

In the model built using data after 1995(Table 3), 55 of the 123 cases (44.72%) were included. We see a similar relationship as the model for all years. Due to low number of cases for all ethnicities in the 55 cases reported after 1995, we could not utilize the full ethnicity variable for analysis. We re-structured the variable to consider Asian and Pacific Islanders versus all other ethnicities as that is the ethnicity that appeared as a risk factor in the first model. Again, the relationship between birth location and type of leprosy was not statistically significant (aOR: 0.11; 95% CI: 0.01–1.35) in this smaller model when controlling for age, sex, and being ethnically Asian or Pacific Islander. However, MB leprosy was still statistically associated with Asian and Pacific Islander ethnicity (aOR: 8.67; 95% CI: 1.30–57.74). Additionally, age as a continuous variable, was associated with PB leprosy during this time range (aOR: 0.91; 95% CI: 0.86–0.98). Given the potential interaction with location of birth and ethnicity, interaction was assessed and there were no interactive effects found in this model.

**Table 3:** Logistic regression model for cases from 1995 to 2018.

	Univariate Analysis		Multivariate Analysis	
Parameter	OR (95% CI)	p-value	aOR (95%)	*p*-value
Birth location				
Domestic	1.0		1.0	
Abroad	1.38 (0.25–7.53)	0.711	0.11 (0.01–1.35)	0.084
Sex				
Female	1.0		1.0	
Male	0.58 (0.16–2.14)	0.41	0.45 (0.10–2.01)	0.295
Ethnicity				
All other ethnicities	1.0		1.0	
Asian or Pacific Islander	4.50 (1.01–19.96)	0.048	8.67 (1.30–57.74)	0.026*
Age	0.95 (0.90–0.99)	0.023	0.91 (0.86–0.98)	0.007*

* significant *p*-value at *p* < 0.05.

aOR = adjusted odds ratio

## Discussion

This novel analysis of leprosy cases in the state of Georgia revealed several notable findings. The younger average age of the population born abroad compared to the domestically born cases is of note, as it shows the greater risk of infection younger populations face in their home countries. Given the higher prevalence of leprosy in many countries of origin for foreign-born cases as compared to the U.S., this makes sense[8]. The increasing trend in both number of MB cases and foreign-born cases after the 1970s suggests the possibility of more leprosy transmission in the region, although enhancements in surveillance, increased immigration to the state, or simply population size may be other explanations. Interestingly, our study found the frequency of MB and PB among domestic born cases compared to cases born abroad to be almost equal (PB: 30.77% vs. 36.90%; MB: 69.23% vs. 63.10%), which is approximately the same split seen in prior studies[11, 19]. The literature shows varying proportions of MB versus PB depending on the country, therefore, we expected a difference between U.S. born and those from other countries, perhaps related to differing propensities towards MB vs PB genetically or from other factors like nutritional deficiencies[23, 24].

The fact that those of Asian and Pacific Islander ethnicity are at greater odds of MB disease is of note. One explanation could be that there is a higher likelihood of MB disease in Asian and Pacific island countries, suggesting factors such as host genetics, micronutrient deficiencies, the limited gene pool of an island nation, or other co-morbidities that depress the immune response to *M*. *leprae*[24]. These immigrant groups may be, thus, at higher risk of secondary transmission of leprosy in their communities, given that MB is more infectious than PB. While little is known about the relationship between specific ethnic groups in the U.S. and leprosy, in one study on Micronesian and Marshallese people living in the United States, MB was more prevalent (approximately 75%) than PB[25]. Additionally, a case analysis of leprosy patients in Toronto, Canada showed similar findings for MB being more prevalent in southeast Asian immigrants[26]. However, there is no specific identifiable literature that explains why this ethnic group would have greater odds of having multibacillary disease. In our study, the adjusted odds ratio for this variable in the model for all years had large confidence intervals (aOR: 5.71; 95% CI: 1.25–26.29) suggesting some uncertainty of the measure given the low study numbers. However, this finding is still important as understanding what ethnic groups are at higher risk for MB assists public health officials in focusing targeted interventions.

In the model built using data after 1995, birth location was again not associated with type of leprosy (aOR: 0.11; 95% CI: 0.01–1.35), but being Asian and Pacific Islander still showed a statistically significant association with MB leprosy (aOR: 8.67; 95% CI: 1.30–57.74). Additionally, age was shown to have a statistically significant relationship with PB leprosy, but with the aOR so close to 1, this is likely not clinically meaningful (aOR: 0.91; 95% CI: 0.86–0.98). Also, in the past 10 years, there has been more evidence of armadillo reservoirs of infection including the southern U.S., Mexico, and even in Brazil, which is the country with the second highest number of cases globally[4, 5, 15–17, 27–34]. Therefore, it is possible that there has been an increase in armadillo associated infections in the Americas in more recent times, even in those that are not U.S. born.

There are several limitations to this study. While leprosy is a reportable disease in Georgia, there is always the potential for underreporting of cases. We would assume that the surveillance data is a complete population of those that have leprosy living in Georgia, but there are likely missed cases due to missed data entry on surveillance reports, individuals who are misdiagnosed and therefore not reported, or those who do not seek care. With such a small number of cases, the loss of 15 cases due to incomplete data also could have significantly impacted the results. The overall small study sample could limit the ability to discover a significant association.

There were several variables that we would have liked to include in the model, but were unable to attain data on. First, we would have liked to know about potential prior armadillo contacts and contact with known infected leprosy patients. While it is not known how armadillos transmit infection, contact with armadillos has been found to be associated with Hansen’s disease[4, 5, 15–17, 27, 28, 31, 33, 34]. We also would have liked to control for socioeconomic status (SES), as it can be theorized that individuals with lower SES may live in more crowded conditions, which is known to be associated with increased cases of leprosy, or potentially could be related to factors that predispose an individual to having MB leprosy. Ideally we would have been able to control for location of birth by country as opposed to a simple dichotomy, but many surveillance reports were incomplete and only indicated the ethnicity and the date the patient immigrated to the United States, not including the country of origin.

Because the sample used is only of cases from Georgia, we cannot generalize these findings to leprosy cases from other states. A more complete study should be done utilizing all cases from the 50 states to better assess the relationship between birth location and type of leprosy diagnosed. This would also give a more holistic view of the epidemiology of leprosy in the United States. Unfortunately, due to limitations in data collection and data use, we were unable to utilize data from all states.

As it stands, leprosy research, specifically research that focuses on United States leprosy patients, is fairly limited. In an increasingly globalized world where movement of people is easier now than ever before, leprosy cases will continue to appear all around the world. The upward trend of cases seen since the 1970s in Georgia and the increasing rates of immigration into the state are an impetus for further study of this reemerging disease which can cause severe disability and around which persistent stigma exists[35]. Although leprosy does not pose a public health threat to the general U.S. population, unique analyses such as this study can have clinical significance and would increase our understanding of the disease. These further studies would allow for more targeted treatment and prevention strategies to help achieve the disease elimination goals.

## Supporting information

S1 File Hansen’s Disease (Leprosy) Surveillance Form. (PDF)
