# Supplementary material for: Trends of leprosy and multibacillary infection in the state of Georgia since the early 1900s
Source: PLoS Negl Trop Dis. 2019 Oct 11;13(10):e0007713. doi: 10.1371/journal.pntd.0007713 (PMC6808490; doi:10.1371/journal.pntd.0007713)
Supplement: S1 File — (PDF) [file pntd.0007713.s001.pdf]

Instructions for Completing the
Hansen's Disease *(Leprosy)* Surveillance Form

The Hansen's Disease or Leprosy Surveillance Form *(LSF)* is the document used to report leprosy cases to the U.S. National Hansen's Disease Registry. These data are used for epidemiological, clinical, and basic research studies throughout the National Hansen's Disease Program *(NHDP),* and are the official source for information on leprosy cases in the U.S. **Please report this case to your state health department. The NHDP does not report to state health departments.**

The information requested on the LSF is used by many clinicians and researchers, and collection of all information is highly desirable. However, the fields that are **boldfaced** on the form and in the instructions below are considered to be the minimal information needed to register a patient. Failure to provide this information will result in the form being returned which creates additional work and may cause delays in obtaining program services for the patient.

1. **Reporting State:** Use the abbreviation of the state from which the report is being sent. This is usually the state of the clinician's office and not necessarily the patient's resident state.
2. **Date of Report:** This is date of the initial LSF completion. If patient was previously reported and has relapsed, write the word "RELAPSE" next to the date.
3. Social Security Number: Optional; self-explanatory.
4. **Patient Name:** Self-explanatory.
5. **Present Address:** Please include the county and zip code which are used to geographically cluster patients.
6. **Place of Birth:** Include state and city, if born in the U.S., or the country, if foreign born.
7. **Date of Birth/Sex:** Self-explanatory.
8. **Race/Ethnicity:** This information should be voluntarily provided by the patient. If the patient refuses or indicates a race/ethnicity category not listed, check the "Not Specified" box.
9. **Date Entered the U.S.:** For patients who have immigrated to the U.S., provide the month and year of entry.
10. Date of Onset of Symptoms: This information is usually the patient's recollection of when classic leprosy symptoms *(rash, nodule formation, paresthesia, decreased peripheral sensation, etc.)* were first noticed.
11. **Date Leprosy First Diagnosed:** Provide the month and year a diagnosis was made. This usually coincides with a biopsy date if one was performed.
12. **How many doctors have you seen for this problem?** This will be based primarily on the patient's recollection. Include the physician reporting the case.
13. **Initial Diagnosis:** Was the patient diagnosed in the U.S. or outside the U.S.
14. **Type of Leprosy:** Classify the diagnosis based on one of the ICD-10-CM diagnosis codes. (NHDP Clinic physicians: Please circle specific classification, if possible)

    A30.5 Lepromatous Leprosy *(macular, diffuse, infiltrated, nodular, neuritic – includes Ridley-Jopling [RJ], Lepromatous [LL] and* A30.4 Borderline lepromatous *[BL]*): A form marked by erythematous macules, generalized papular and nodular lesions, and variously by upper respiratory infiltration, nodules on conjunctiva or sclera, and motor loss.
    A30.1 Tuberculoid Leprosy *(macular, maculoanesthetic, major, minor, neuritic – includes RJTuberculoid [TT] and* A30.2 Borderline tuberculoid *[BT])*: A form marked by usually one lesion with well-defined margins with scaly surface and local tender cutaneous or peripheral nerves.
    A30.0 Indeterminate *(uncharacteristic, macular, neuritic)*: A form marked by one or more macular lesions, which may have slight erythema.
    A30.3 Borderline *(dimorphous, infiltrated, neuritic – includes RJ Borderline [BB] or true mid disease only)*: A form marked by early nerve involvement and lesions of varying stages.
    A30.8 Other Specified Leprosy: Use this code when the diagnosis is specified as "leprosy" but is not listed above *(A30.0-A30.3)*, including 'pure neural' disease.
    A30.9 Leprosy, Unspecified: Use this code when the diagnosis is identified as "leprosy" but inactive.
15. Diagnosis of Disease: Reaction=Y if steroids required. Enter INITIAL biopsy and skin smear dates and results.
16. **Residence *(Pre-diagnosis)*:** List all cities, counties, and states in the U.S. and all foreign countries a patient resided in BEFORE leprosy was diagnosed. This information is used to map all places where U.S. leprosy cases have resided.
17. **Disability: Eye, Hand & Foot.** For each eye, hand and foot check Yes or No. [Normal always = No]
    Loss of any sensation in hands or feet; for Eyes, is blinking abnormal (very infrequent?). Normal = No
    Visible deformity (muscle wasting, clawing of fingers or toes, ulcers or other abnormality of the hands or feet. For Eyes, lagophthalmos or reduced vision (e.g. cataract). Normal = No
18. Current Household Contacts: Self-explanatory.
19. Current Treatment for Leprosy: Date that treatment started and indicate all drugs used for initial treatment.

FOR NHDP USE ONLY

HANSEN'S DISEASE *(LEPROSY)* SURVEILLANCE FORM

NATIONAL HANSEN'S DISEASE PROGRAM

1770 PHYSICIANS PARK DRIVE

BATON ROUGE, LA 70816

1-800-642-2477

1 Reporting State: ____________

2 Date of Report: ____________

3 Social Security Number *(optional):* ____________

4 Patient Name: *(Last)* ____________ *(First)* ____________ *(Middle)* ____________

5 Present Address: Street ____________ City ____________

County ____________ State ____________ Zip ____________

6 Place of Birth: State ____________ City ____________

Country ____________

7 Date of Birth: ____________

Sex: ☐ Male ☐ Female

8 Race/Ethnicity: ☐ White, Not Hispanic ☐ White, Hispanic ☐ American Indian, Alaska Native ☐ Indian, Middle Easterner ☐ Black, Not Hispanic ☐ Black, Hispanic ☐ Asian ☐ Native Pacific Islander ☐ Not Specified

9 Date Entered U.S.: Mo. ______ Yr. ______

10 Date of Onset of Symptoms: Mo. ______ Yr. ______

11 Date Leprosy First Diagnosed: Mo. ______ Yr. ______

12 How many doctors have you seen for this problem? ☐

13 Initial Diagnosis: ☐ In U.S. ☐ Outside U.S.

14 Type of Leprosy: *(ICD-10-CM Code)* (NHDP Clinic physicians: Please circle specific classification, if possible)

☐ Lepromatous Leprosy *(A30.5 - LL)*
☐ Borderline Lepromatous *(A30.4 - BL)*
☐ Tuberculoid *(A30.1 - TT)*
☐ Borderline Tuberculoid *(A30.2 - BT)*
☐ Indeterminate *(A30.0 - IN)*
☐ Borderline *(A30.3 - BB)*
☐ Other Specified Leprosy *(A30.8)*
☐ Leprosy, Unspecified *(A30.9)*

15 Diagnosis of Disease:

	Yes	No
Leprosy reaction at diagnosis?	☐	☐
Was biopsy performed in U.S.?	☐	☐

Date ____ / ____ / ____

Result ____________

Skin Smear? Yes ☐ No ☐ ____ / ____ / ____ Date

BI: Positive ____ Negative ____

16 List all places in the U.S.A. and all foreign countries a PATIENT resided *(Including Military Service)* BEFORE leprosy was diagnosed:

TOWN	COUNTY	STATE	COUNTRY	INCLUSIVE DATES From Mo./Yr.	To Mo./Yr.

17 Disability:

	Hands Right Yes	Hands Right No	Hands Left Yes	Hands Left No	Feet Right Yes	Feet Right No	Feet Left Yes	Feet Left No
Loss of Sensation?	☐	☐	☐	☐	☐	☐	☐	☐
Visible deformity?	☐	☐	☐	☐	☐	☐	☐	☐

	Eyes Right Yes	Eyes Right No	Eyes Left Yes	Eyes Left No
Blink abnormal?	☐	☐	☐	☐
Lagophthalmos?	☐	☐	☐	☐

18 Current Household Contacts: Name/Relationship

1 ____________

2 ____________

3 ____________

4 ____________

19 Current Treatment for Leprosy: *(check all that apply)*

Date Treatment Started: ____ / ____ (Mo. / Yr.)

☐ Dapsone ☐ Rifampin ☐ Clofazimine

☐ Other *(list)* ____________

20 Name and Address of Physician: ____________

Investigator:

This form may be FAXED to NHDP at (225) 756-3706

A copy of this form should be sent to your local or state health department.
